# Behavioral Selectivity: Species-Specific Effects of Nutmeg, Cinnamon, and Clove Essential Oils on *Sitophilus oryzae* and Its Parasitoid *Lariophagus distinguendus*

**DOI:** 10.3390/molecules30173627

**Published:** 2025-09-05

**Authors:** Prangthip Parichanon, Roberta Ascrizzi, Guido Flamini, Ylenia Pieracci, Maria Cristina Echeverría, Sania Ortega-Andrade, Barbara Conti

**Affiliations:** 1 Department of Agriculture, Food and Environment, University of Pisa, Via del Borghetto 80, 56124 Pisa, Italy; barbara.conti@unipi.it; 2 Department of Pharmacy, University of Pisa, Via Bonanno 6, 56126 Pisa, Italy; roberta.ascrizzi@unipi.it (R.A.); guido.flamini@unipi.it (G.F.); ylenia.pieracci@agr.unipi.it (Y.P.); 3 Nutrafood Research Center, University of Pisa, Via del Borghetto 80, 56124 Pisa, Italy; 4 Networked Science Research Group (eCIER), Department of Biotechnology, Technical University of the North, Av. 17th of July 5–21 & Gen. Jose Maria Cordova, Ibarra 100150, Ecuador; mc.echeverria@hotmail.com (M.C.E.); smortega@utn.edu.ec (S.O.-A.)

**Keywords:** rice weevil, behavioral responses, multi-trophic interactions, repellency, attractiveness, stored-product pest

## Abstract

The integration of essential oils (EOs) with biological control agents offers a promising alternative to synthetic pesticides, though compatibility remains unclear. This study evaluated nutmeg (*Myristica fragrans*, NM), cinnamon (*Cinnamomum verum*, CIN), and clove (*Syzygium aromaticum*, CL) specifically on *S. oryzae* and *L. distinguendus*. Olfactory and behavioral responses to whole EOs and major constituents (myristicin, cinnamaldehyde, eugenol) were analyzed using the area preference method (APM) and two-choice behavioral bioassay (TCB), with confirmation by gas chromatography–mass spectrometry (GC-MS). In *S. oryzae*, APM showed attraction to all three EOs (PI = 0.14 to 0.56). A paradox emerged, however, as single constituents were mostly repellent (eugenol: PI = −0.58 to −0.70; cinnamaldehyde: PI shifted from 0.16 to −0.20), underscoring the complexity of EO mixtures where multiple compounds act jointly rather than individually. In contrast, *L. distinguendus* strongly avoided CL and CIN in TCB, with fewer than 30% of parasitoids choosing the EO-treated side (χ^2^ test, *p* < 0.05). CIN therefore demonstrated selective potential, simultaneously attracting *S. oryzae* while repelling *L. distinguendus*. These findings highlight the dual role of EOs as botanical pest control tools, while stressing the need to consider non-target effects before practical application.

## 1. Introduction

Cereal grains such as rice, wheat, maize, and barley are fundamental to the global food system, serving as primary staples for both human consumption and livestock feed [1,2,3]. Among the major threats to stored cereals is the rice weevil, *Sitophilus oryzae* (Linnaeus) (Coleoptera Curculionidae), one of the most destructive storage pests worldwide [4,5]. By completing development inside kernels, infestations often remain undetected until severe internal damage has occurred, resulting in substantial postharvest losses [6,7,8]. Favorable storage conditions such as high temperature and humidity further accelerate population growth, making management increasingly difficult [9,10,11].

Conventional control relies largely on synthetic insecticides; however, these treatments face significant limitations due to resistance development, residue accumulation in food products, and risks to human health and non-target organisms [12,13]. As a result, biological control strategies have gained growing interest. The parasitoid wasp *Lariophagus distinguendus* (Förster) (Hymenoptera Pteromalidae) has proven particularly effective, parasitizing the larval stage of *Sitophilus* spp. inside grains and reducing pest populations without leaving chemical residues [14,15,16]. Although parasitoids such as *L. distinguendus* can effectively reduce *S. oryzae* populations, their success depends on compatibility with other control methods [17]. One potential approach involves the use of essential oils (EOs), whose strong olfactory activity raises concerns that they could either complement or disrupt parasitoid efficiency, underscoring the need to examine their combined use.

Among these methods, EOs have attracted particular attention due to their volatility, low persistence, and minimal toxicity to vertebrates. Depending on the target species and concentration, they can act as repellents, attractants, or insecticides [18,19], making them suitable for postharvest use and, in some cases, obtainable from agricultural by-products in support of circular economy approaches [20,21,22]. Yet, their dual effects could also influence beneficial parasitoids in unexpected ways, raising concerns about compatibility with biological control.

EOs are known to exert species- and context-specific behavioral effects [23,24,25]. While their efficacy against *S. oryzae* has been well documented, much less is understood about their impact on natural enemies such as *L. distinguendus*. This knowledge gap limits our ability to evaluate potential non-target impacts—an essential step toward designing integrated, selective strategies that exploit essential oil (EO) activity without undermining parasitoid-mediated control in cereal storage systems.

Olfactory cues play a central role in host-location behavior among parasitic Hymenoptera. Several classes of plant-derived compounds—such as phenylpropanoids including eugenol and cinnamaldehyde—have been implicated in modulating insect olfactory pathways [26,27,28]. Although direct evidence in *L. distinguendus* is limited, these compounds may potentially affect parasitoid foraging behavior through mechanisms related to olfactory perception. Gaining a deeper understanding of species-specific behavioral responses can support the development of targeted EO-based applications that enhance, rather than disrupt, biological control.

Ultimately, our goal is to advance EO-based biological control strategies suitable for organic and low-input cereal production systems. To this end, we examined the behavioral effects of three commonly used EOs—nutmeg (*Myristica fragrans* Houtt., NM), cinnamon (*Cinnamomum verum* J. Presl, CIN), and clove (*Syzygium aromaticum* (L.) Merr. & L. M. Perry, CL)—on both *S. oryzae* and its parasitoid *L. distinguendus*. Although these EOs have demonstrated strong bioactivity against cereal pests, their roles in pest–parasitoid interactions remain poorly characterized. We hypothesize that the selected EOs and their major constituents elicit species-specific behavioral responses, deterring the target pest while minimizing interference with the parasitoid activity. To explore their potential for selective application, we employed two complementary behavioral assays: two-choice behavioral bioassay (TCB), which assesses directional preference, and area preference method (APM), which measures surface-level behavioral responses. This dual-assay approach enables a more nuanced evaluation of EO-driven behaviors across trophic levels.

## 2. Results

### 2.1. Chemical Profiles of Nutmeg, Cinnamon, and Clove Essential Oils

As shown in Table 1, the chemical composition of the three essential oils (EOs) differed markedly. NM contained over 25 identified volatile compounds, representing 99.8% of the total composition. Monoterpene hydrocarbons were the predominant class (58.0%), followed by phenylpropanoids (35.1%). The main constituents among the monoterpene hydrocarbons were α-pinene (17.0%), sabinene (14.1%), and β-pinene (13.4%). In the phenylpropanoid class, myristicin was the principal compound (33.0%), making it a key component of the oil. Minor compounds included elemicin (1.8%) and methyl eugenol (0.2%).

CIN comprised approximately 21 identified compounds, accounting for 100.1% of the total volatiles. Phenylpropanoids dominated the chemical profile (88.5%), with (*E*)-cinnamaldehyde as the major constituent (78.5%), followed by eugenol (5.2%) and (*E*)-cinnamyl acetate (3.8%). Sesquiterpene hydrocarbons (5.7%) and monoterpene hydrocarbons (2.2%) were present in minor proportions, while other non-terpene derivatives and oxygenated monoterpenes were detected in trace amounts (<2%).

CL was characterized by seven chemical constituents, with phenylpropanoids accounting for 95.0% of the composition. Eugenol was the predominant compound (89.2%), along with eugenol acetate (5.8%). The minor constituents included β-caryophyllene (3.9%), α-humulene (0.5%), methyl salicylate (0.3%), caryophyllene oxide (0.2%), and 2-heptyl acetate (0.1%).

### 2.2. Behavioral Responses of S. oryzae and L. distinguendus to Essential Oils in a Two-Choice Olfactometer

**Figure 1:**
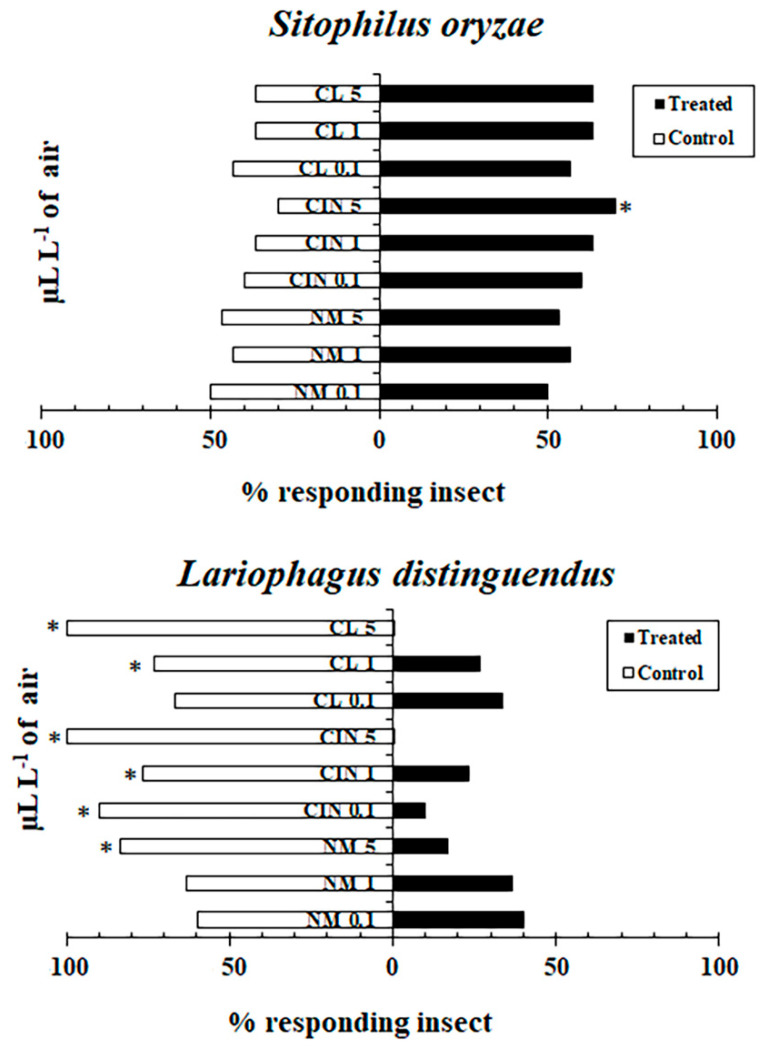
Behavioral responses assessed in a two-choice olfactometer bioassay of (**above**) *Sitophilus oryzae* and (**below**) *Lariophagus distinguendus* to clove (CL), cinnamon (CIN), and nutmeg (NM) essential oils, tested at three concentrations: 0.1% (CL0.1, CIN0.1, NM0.1 = 0.24 µL L^−1^ air equivalent), 1% (CL1, CIN1, NM1 = 2.39 µL L^−1^ air equivalent), and 5% (CL5, CIN5, NM5 = 11.95 µL L^−1^ air equivalent). Asterisks indicate a significant difference from a 50:50 distribution between control and treatment (*, *p* < 0.05; χ^2^ test). Values are means ± SD (*n* = 30 per treatment).

The TCB demonstrated that *S. oryzae* and its parasitoid, *L. distinguendus*, exhibited species-specific and concentration-dependent responses to the volatile fractions of CL, CIN, and NM, when tested at three concentration levels—low, medium, and high (corresponding to nominal vapor-phase concentrations of 0.24, 2.39, and 11.95 µL L^−1^ air equivalent; Figure 1). Since EOs are complex mixtures, it should be noted that more volatile components will likely have dominated the headspace in our assays, which means that the vapor phase to which insects were exposed may not be fully representative of the composition of the original oil.

For *S. oryzae*, all three EOs triggered attraction at each concentration tested, with the most pronounced response observed for CL at the highest concentration (11.95 µL L^−1^ air equivalent), where a significant preference for the treated chamber was observed (χ^2^ test, *p* < 0.05). By contrast, *L. distinguendus* generally avoided the EO volatiles, especially CL and CIN at medium and high concentrations, as evidenced by a significantly greater number of individuals choosing the control chambers (*p* < 0.05). However, *L. distinguendus* responses to NM were generally neutral at low and medium concentrations, while a significant repellence was observed at the highest concentration tested (NM5; 11.95 µL L^−1^ air equivalent, χ^2^ test, *p* < 0.05).

### 2.3. Behavioral Responses of S. oryzae and L. distinguendus to Essential Oils in the Area Preference Method

The behavioral responses of *S. oryzae* to NM, CIN, and CL were evaluated using the APM at three vapor-phase concentrations (Table 2). In this assay, all three EOs elicited positive PI values at every concentration tested (0.94, 1.89, and 3.77 µL L^−1^ air equivalent), indicating attractive effects. APM results are reported only for *S. oryzae*, as discussed in materials and methods (Section 4.5) since *L. distinguendus* is a flying species and therefore not suitable for testing in Petri dishes. Notably, the preference index (PI) values for CL ranged from 0.40 ± 0.21 to 0.56 ± 0.11, which were the highest among the tested EOs. Duncan’s multiple range test further indicated that CL and CIN formed a significantly different group from NM across concentrations. Thresholds for PI interpretation were adopted from Lacotte et al. [30], where values between 0.1 and 0.4 are considered moderate and values ≥ 0.4 indicate strong attraction.

The results from the TCB were generally in line with the APM findings. Both APM and TCB revealed that CIN and CL elicited stronger attraction than NM, particularly at higher concentrations. Despite some variation in significance thresholds, the dual assays showed consistent behavioral patterns, reinforcing the robustness of EO-induced attraction.

### 2.4. Bioactivity of Major EO Constituents Against S. oryzae Using the Area Preference Method

As shown in Table 3, among CIN components, cinnamaldehyde exhibited a concentration-dependent shift from attraction at the lowest concentration PI = 0.16 ± 0.24 at 0.94 µL L^−1^ air equivalent) to repellency at the highest concentration (PI = −0.20 ± 0.16 at 3.77 µL L^−1^ air equivalent). Meanwhile, α-terpineol were consistently repellent across all doses tested (PI = −0.48 ± 0.31 to −0.50 ± 0.07), while 1,8-cineol was neutral at lower concentrations and became repellent only at the highest dose (PI = −0.14 ± 0.11 at 3.77 µL L^−1^ air equivalent). For CL, the major constituent eugenol displayed significant repellency at all concentrations (PI = −0.58 ± 0.33 to −0.70 ± 0.10), while β-caryophyllene, present in both CIN and CL showed neutral at the lowest concentration (PI = 0.02 ± 0.50) to repellency as the dose increased (PI = −0.16 ± 0.23 at 1.89 µL L^−1^ and −0.24 ± 0.17 at 3.77 µL L^−1^). In NM, several constituents elicited attractive responses at lower concentrations. For example, β-pinene, α-pinene, and limonene produced attraction at 0.94 µL L^−1^ air equivalent (PI = 0.40 ± 0.34, 0.38 ± 0.18, and 0.20 ± 0.10, respectively), became neutral at 1.89 µL L^−1^ (PI = −0.02 ± 0.18, 0.04 ± 0.62, and 0.08 ± 0.23), and turned repellent at 3.77 µL L^−1^ (PI = −0.18 ± 0.43, −0.26 ± 0.15, and −0.20 ± 0.07). Similarly, myristicin elicited an attractive response at low dose (PI = 0.73 ± 0.12 at 0.94 µL L^−1^ air equivalent), which decreased to neutrality (PI = 0.10 ± 0.26 at 1.89 µL L^−1^ air equivalent) and became to repellency at higher concentrations (PI = −0.33 ± 0.25 at 3.77 µL L^−1^ air equivalent). In contrast, other NM constituents such as sabinene, terpinolene, linalool, and methyl eugenol showed clear dose-dependent repellency, especially at 3.77 µL L^−1^ air equivalent.

MR-08 (menthol propylene glycol carbonate), a substance permitted for use in food contact applications, was employed as a positive control due to its well-documented repellent efficacy against *S. oryzae* and its established use for preventing insect infestations in food storage and preparation environments [31]. As expected, MR-08 produced significant repellent effects at all tested concentrations (PI = −0.18 ± 0.26, −0.26 ± 0.09, and −0.42 ± 0.19 at 0.94, 1.89, and 3.77 µL L^−1^ air equivalent, respectively), further supporting the reliability of the APM assay.

Overall, these results indicate that the individual EO constituents contribute distinct and sometimes opposing behavioral effects compared to the complete EO. Notably, eugenol in CL and cinnamaldehyde in CIN were mainly responsible for the repellent responses, whereas myristicin, β-pinene, and other monoterpenes in NM may underlie its attractive properties at lower doses.

## 3. Discussion

This study demonstrates that the tested EOs can act as both attractants and repellents toward *S. oryzae* and its parasitoids *L. distinguendus*. By employing a dual-assay approach—including both TCB and APM—we were able to compare the behavioral trends observed in the two tests under different experimental conditions. While the APM primarily evaluates insect preference for treated versus untreated surfaces with volatile compounds, the TCB measures directional olfactory responses to EO volatiles. The combined use of these methods allowed for a broader interpretation of EO-driven behavioral patterns in the studied species [23,24].

This work is, to the best of our knowledge, one of the first to simultaneously investigate the behavioral effects of EOs on both a key stored-product pest and its natural parasitoid through a dual-assay approach. These findings provide important new evidence for the species-specific action of EO-based strategies in postharvest pest management.

Our findings revealed that whole EOs of CL and CIN consistently attracted *S. oryzae*, whereas their dominant constituents—eugenol and cinnamaldehyde—were repellent when tested individually in the APM, with cinnamaldehyde showing attraction only the lowest concentration. This discrepancy highlights the importance of evaluating EOs as complex mixtures rather than isolated compounds [24,31]. Such outcomes may arise from olfactory masking or synergistic interactions among volatiles, where minor constituents such as β-caryophyllene modulate the perception of dominant odorants [15,32]. Non-linear neutral processing in insect olfaction may further explain why whole oils elicit behavioral responses not predictable from single components [33,34,35,36].

This apparent paradox, where whole EOs elicited attraction but their dominant constituents were largely repellent, underscores the complexity of EO mixtures. Similar discrepancies have been reported in other systems: Akotsen-Mensah et al. [37] showed that blends of host-plant volatiles attracted *Halyomorpha halys* nymphs even when single compounds did not; Ahmed et al. [38] demonstrated that binary combinations of black pepper, eucalyptus, rosemary, and tea tree EOs caused significantly higher mortality in *Myzus persicae* than single oils, with clear synergistic interactions; and Filomeno et al. [39] emphasized the role of constituent interactions in shaping the bioactivity of *Eucalyptus* EO against *R. dominica*. Together, these examples support the view that EO bioactivity must be interpreted as an emergent property of mixtures rather than a simple sum of individual effects. Future studies should therefore include systematic testing of reconstituted EO blends to disentangle the relative contribution of single constituents and their interactions.

Even if the context and presentation of EO odors can influence insect responses [8] in our experiment the outcome of the two behavioral tests (TCB and APM) using whole oils is of absolute attractiveness to *S. oryzae*, as evidenced by the APM results and by the general trend in TCB, even if not all individual data are statistically significant.

Of particular note, our study found clear species-specific responses: *S. oryzae* was attracted by the whole EOs whereas its parasitoid, *L. distinguendus*, generally avoided these volatiles, especially at higher concentrations. Interestingly, CIN significantly attracted *S. oryzae*, while at the same concentration (5%, 11.95 µL L^−1^ air equivalent) it repels significantly *L. distinguendus*. This selective effect represents a promising basis for the development of more targeted pest management tools. Similar patterns of species-specific selectivity have been observed with other EOs, with some products showing efficacy against pests while having comparatively lower impact on natural enemies; however, non-target effects can still occur and should be evaluated [40,41]. Such species-specific selectivity may arise from differences in olfactory perception and processing, which underlie the diversity of odor-mediated behaviors across insect species [42]. While *S. oryzae* may use a broader range of volatiles—including those from grains and spices—for host location and aggregation [24,43], parasitoids like *L. distinguendus* may have evolved to focus on host- or host-damage-related cues, potentially making them repelled by high concentrations of certain plant volatiles [44]. These findings also highlight the potential for the development of push–pull strategies in stored grain systems, where selective attractants and repellents could be combined to optimize pest management while conserving beneficial parasitoids.

Concentration is also a critical factor: while certain EO constituents act as repellents at higher doses, their presence in lower concentrations within complex blends may result in neutral or even attractive responses—a biphasic (hormetic) effect also reported in previous studies [23,29,43,45]. Such dynamics underscore the need for careful optimization of EO formulations and delivery systems in storage environments [25]. Emerging technologies, such as controlled-release dispensers and smart delivery systems, could be explored to maintain effective EO concentrations over time while minimizing non-target impacts [46].

Furthermore, the use of attractive EOs must be carefully managed in practice. CIN, for example, has shown the most promising selectivity in TCB as an attractant and can be used to trigger traps for the mass trapping of pest populations, or in push-pull techniques where the pest is attracted and the parasitoid repelled [47,48,49]. In line with regulatory standards, potential non-target effects, such as the impact on other natural enemies or conservative arthropods, as well as EO residue and food safety issues, should also be evaluated [21,50].

It is noteworthy that the observed differences in behavioral responses between assays could partly result from the differences in exposure methods and concentrations, as the APM involved both olfactory and contact exposure, necessitating lower maximal concentrations compared to the olfactometer assay.

Our findings demonstrate some variations between assays, particularly the APM and TCB. Although CL showed slightly higher preference index values in the APM, the overall selectivity observed in TCB towards CIN was considered more robust due to the additional repellency to parasitoids and clearer consistent trend. Such assay-dependent variability emphasizes the importance of employing multiple complementary methods when assessing EO-induced behaviors [51].

In summary, our findings highlight the three EOs tested and, in particular, CIN as a promising tool for species-selective biological control of *S. oryzae* and its parasitoid interactions in stored cereals. The dual-assay approach applied here provides a robust framework for evaluating botanical solutions that can suppress pest populations while conserving beneficial parasitoids. Future research should prioritize the validation of these selective effects under realistic storage conditions and employ functional genomics and electrophysiological methods to unravel the molecular basis of species-specific responses. In this context, odorant-binding proteins and odorant receptors represent particularly promising molecular targets, as both classes of proteins are increasingly explored for their roles in semiochemical perception and their potential use in the rational design of new pest control agents [52,53].

## 4. Materials and Methods

### 4.1. Insect Rearing Conditions

#### 4.1.1. *Sitophilus oryzae*

The *S. oryzae* population used in this study originated from a laboratory colony maintained for over 10 years at the Entomology Laboratory of the Department of Agriculture, Food and Environment, University of Pisa, without any history of insecticide exposure. The rearing method was adapted from Abenaim et al. [31]. The insects were reared in 5 L plastic containers (29 × 18 × 9 cm) with netted lids. Each container was filled with 500 g of organic whole-grain barley (EcorNaturasì SpA, San Vendemiano, Italy) with a moisture content of approximately 12–14%, which had been previously frozen at −20 °C for one week to eliminate any potential pests. Rearing for trials was initiated with approximately 200 unsexed adults and maintained under controlled conditions (25 ± 1 °C, 60–70% relative humidity (RH), and constant darkness). The insects were transferred to fresh containers with new grain every three months. To obtain adults of uniform age for bioassays, newly emerged individuals were separated from the rearing by daily sieving (mesh size 2.0 mm). These adults were then grouped in a new container for 5–7 days with approximately 20 g of fresh barley to ensure sexual maturity before being used in the experiments. Rearing containers were kept isolated from other insect colonies to avoid cross-contamination.

#### 4.1.2. *Lariophagus distinguendus*

The *L. distinguendus* population used in this study was from a laboratory colony maintained continuously for several years at the Department of Agriculture, Food and Environment, University of Pisa. Parasitoids were reared using their natural host, *S. oryzae*. To obtain host larvae, 50 g of disinfested organic whole-grain barley (approximately 12–14% moisture content) was placed into a 1 L glass jar (12 cm height × 9 cm diameter). Twenty unsexed *S. oryzae* adults were introduced into each jar and allowed to oviposit for five days. After removing the host adults, the jars were incubated at 25 ± 1 °C and 60–70% RH. When the host larvae were 14–21 days old, 2–10 mated female *L. distinguendus* (2–3 days old) were introduced into each jar. The jars, covered with fine-mesh cloth to prevent escape, were maintained under the same incubation conditions, including constant darkness. Newly emerged adult parasitoids (15–20 days post-parasitization) were collected daily using an aspirator and used for the bioassays within 24 h of emergence. No supplemental feeding was provided before testing.

### 4.2. Essential Oils and Chemical Standards

Three EOs were selected for this study based on their reported bioactivity, commercial availability, and cost. The EOs of NM and CIN were purchased from Biokyma (Anghiari, Italy) and Sigma-Aldrich S.r.l. (Milan, Italy), respectively. The EO of CL was obtained from flower buds collected in July 2024 from cultivated trees at Universidad Técnica del Norte, Ibarra, Ecuador (0°21′28″ N, 78°7′36″ W, altitude 2200 m). The plant material was air-dried at room temperature (~25 °C) in a light-protected environment at the Department of Biotechnology, Universidad Técnica del Norte, and ground into a fine powder. For CL, 100 g of dried flower bud powder was extracted by hydrodistillation using a Clevenger apparatus for 3 h. The resulting EO was dried over anhydrous sodium sulfate and stored in glass ampoules at 4 °C until further analysis.

The following chemical standards, corresponding to major constituents identified in the EOs, were purchased from Sigma-Aldrich S.r.l. (Milan, Italy): cinnamaldehyde, 1,8-cineole, α-terpineol, eugenol, β-caryophyllene, sabinene, β-pinene, α-pinene, myristicin, limonene, terpinolene, linalool, and methyl eugenol.

All EOs were stored in amber glass vials at 4 °C until use. The commercial EOs and chemical standards used in the assays were of analytical grade, with purities ≥ 98%, as specified by the suppliers.

### 4.3. Gas Chromatography–Mass Spectrometry (GC-MS) Analysis

For the analysis of chemical composition, each EO was diluted to 5% in High-Performance Liquid Chromatography-grade *n*-hexane. The analysis was performed using an Agilent 7890B gas chromatograph coupled to an Agilent 5977B mass selective detector (Agilent Technologies, Santa Clara, CA, USA). The system was equipped with an HP-5 MS capillary column (30 m × 0.25 mm i.d., 0.25 µm film thickness). The GC-MS oven temperature was programmed to start at 60 °C, hold for 1 min, then increase to 240 °C at a rate of 3 °C/min, and hold for 5 min. Helium was used as the carrier gas at a constant flow rate of 1 mL/min. The injection volume was 1 µL with a split ratio of 1:25. The injector and transfer line temperatures were set at 220 °C and 240 °C, respectively. The mass detector was operated in electron ionization mode at 70 eV. Mass spectra were recorded in full scan mode over a mass range of *m*/*z* 30–300. The identification of constituents was performed by comparing their retention times and calculated linear retention indices (LRI) with those of authentic standards, a C_6_–C_25_ *n*-hydrocarbon series, and with mass spectra from the NIST [54] and Adams [29] libraries. The relative percentage of each component was calculated by peak area normalization.

### 4.4. Two-Choice Olfactometer Tests

**Figure 2:**
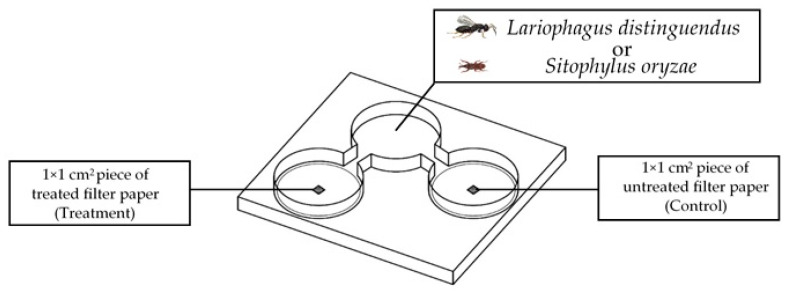
Diagram of the Two-Choice Behavioral Bioassay (TCB) used to test the olfactory responses of *Sitophilus oryzae* and its parasitoid *Lariophagus distinguendus* to EOs. Individual insects were released into a central chamber and allowed to choose between a control and EO stimulus, each presented on a square piece of filter paper in opposing chambers.

To evaluate whether the selected EOs (NM, CIN, and CL) acted as attractants or repellents, we examined the behavioral responses of *S. oryzae* and its parasitoid, *L. distinguendus*, utilizing a two-choice olfactometer. This procedure was conducted in accordance with the methodology of Parichanon et al. [23] (Figure 2).

The olfactometer consisted of a clear plastic arena (15 × 15 × 1 cm) with three circular chambers (4.0 cm in diameter; 12.56 cm^3^ volume in total) connected by straight channels. One side chamber served as the control and contained a 1 × 1 cm piece of filter paper (Whatman No. 1, Cytiva, Maidstone, UK) treated with 3.0 µL of pure ethanol. The opposite chamber held an identical paper treated with 3.0 µL of an EO diluted in ethanol at 0.1%, 1%, or 5%, yielding estimated vapor-phase concentrations equivalent to 0.24, 2.39, and 11.95 µL L^−1^ air equivalent, respectively. Filter papers were air-dried under a fume hood for 10–15 min before testing to ensure complete solvent evaporation and avoid non-specific effects.

Each test began by introducing a single unsexed adult insect into the central chamber. The arena was sealed with a glass plate, and insect movement was observed for up to 6 min. A response was considered valid if the insect entered one of the lateral chambers within 20 s and remained there for at least 30 s. Individuals that failed to make a choice within the allotted time were excluded from the analysis.

For each EO concentration, three replicates consisting of 10 valid individual responses were collected, giving a total of *n* = 30 per treatment. The entire experiment was independently repeated three times. All trials were conducted in a controlled environment (25 ± 1 °C, 65% RH) under constant illumination (Philips 30 W/33 fluorescent lamp; 10,000 lux, Amsterdam, the Netherlands). To minimize positional bias, the arena was rotated 90° after each test. Filter papers were replaced between trials, and the olfactometer was cleaned with hexane followed by distilled water every four runs.

### 4.5. Bioactivity Assessment of EOs and EO Components Against S. oryzae Using the Area Preference Method

**Figure 3:**
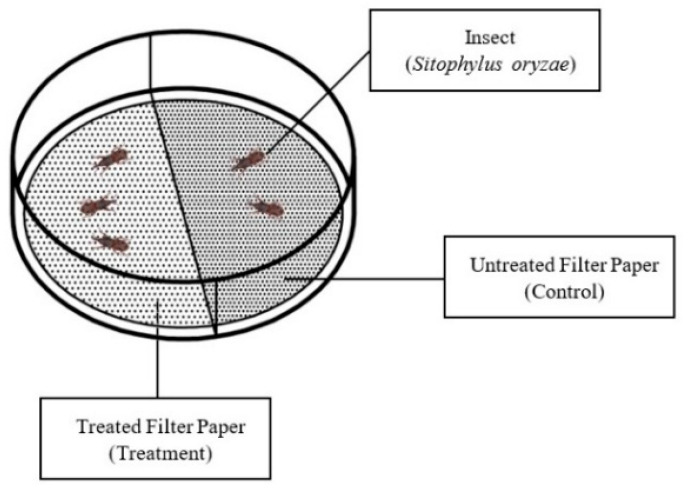
Schematic representation of the Area Preference Method (APM) used to evaluate the behavioral responses of *Sitophilus oryzae* to EOs and main compounds. A treated and an untreated semicircle of filter paper were placed inside a Petri dish, allowing insects to move freely. Insect position was recorded after 24 h to calculate the Preference Index (PI).

The selection of major EO constituents for testing—including cinnamaldehyde from CIN, eugenol and β-caryophyllene from CL, and myristicin, and other monoterpenes from NM—was based on their high relative abundance as identified by GC–MS profiling (Table 1), along with their known or suspected bioactivity in stored-product pest systems. These compounds represent the primary bioactive volatiles in the respective EOs and were selected to clarify their individual contributions to the behavioral responses observed with the complete EOs. The bioactivity of both the individual components and the three complete EOs (CIN, CL, and NM) against adult *S. oryzae* was evaluated using the APM, following a modified protocol based on Parichanon et al. [23] (Figure 3). The APM was only conducted with *S. oryzae*, as *L. distinguendus* is a flying species and cannot be reliably contained in the Petri dish. The concentration ranges tested in the APM and TCB assays were similar to capture a broader range of dose-dependent effects but with slight differences due to assay format and technical constraints as reported in previous studies [55].

Each assay was performed in a round glass Petri dish (10.0 cm diameter, 1.35 cm height; 105.98 cm^3^ volume), lined at the bottom with two semicircular sections of Whatman No. 1 filter paper. One side was treated with 500 µL of absolute ethanol as a negative control, while the opposite side was treated with 500 µL of an EO or main compound solution in ethanol at concentrations of 0.02%, 0.04%, and 0.08%, corresponding to 0.94, 1.89, and 3.77 µL L^−1^ air equivalent, respectively. The selected concentrations were based on preliminary dose–response screenings and literature precedent [56,57], and were intended to encompass both sublethal and overtly repellent levels as well as the attractive ones. While the exposure routes differ between APM (contact or surface-level) and TCB (vapor-phase olfactory exposure), similar EO concentrations were used to identify behavioral trends and concentration-dependent effects across assays. Only the higher concentration in TCB namely 11.95 µL L^−1^ air equivalent has been reduced to a quarter thus to 3.77 µL L^−1^ air equivalent as the APM involve also the contact with the EO or the compound. To eliminate solvent effects, treated papers were air-dried under a fume hood for 15 min before insect exposure. Twenty unsexed *S. oryzae* adults were introduced into each arena, and lids were sealed with Parafilm™ to maintain environmental stability. Each treatment was tested in five replicates per concentration and repeated across three independent trials. Petri dishes were then placed in an incubator maintained at 25 ± 1 °C and 65% RH and were covered with an inverted black vase to ensure darkness and reduce phototactic influence. Insect distribution between the two filter paper zones was recorded 24 h after exposure.

Behavioral responses were quantified using a preference index (PI), calculated using the formula:

PI = (nT − nC)/(nT + nC)
(1)

where nT represents the number of insects on the treated filter paper and nC the number on the control side. Positive PI values indicate attraction, negative values reflect repellency, and zero indicates no preference, with insects distributed equally between the two zones. This formulation was adapted from Lee et al. [56], Caballero-Gallardo et al. [57], and further refined in Parichanon et al. [23]. To support interpretation, PI scores were grouped into attractiveness and repellency categories based on thresholds described by Lacotte et al. [29].

### 4.6. Statistical Analysis

For the APM assay, PI values were calculated and expressed as mean ± standard deviation (SD). For the APM assays with whole EOs (Table 2), treatment means were compared using Duncan’s multiple range test (*p* < 0.05). For the APM component-level assays (Table 3), PI values were reported descriptively (mean ± SD) without multiple-comparison statistics; behavioral responses were classified as repellent (PI < −0.11), neutral (0.10 ≤ PI ≤ 0.10), or attractive (PI > 0.11) following the thresholds of Lacotte et al. [29]. For the TCB assay, the proportion of insects selecting the EO-treated chamber was analyzed using likelihood ratio and Pearson’s chi-square tests, under the null hypothesis of an equal (50:50) distribution between treated and control chambers. Statistical significance was established at *p* < 0.05. All analyses were conducted in SPSS version 22.0 (IBM, Armonk, NY, USA).

## 5. Conclusions

This study revealed that the EOs of CL, CIN, and NM can attract *S. oryzae*, even though their individual constituents acted as repellents when tested alone. The repellent activity observed for individual EO constituents suggests that the attraction seen with whole EOs is likely due to interactions among their various constituents, highlighting the importance of evaluating complete EO mixtures rather than isolated components. Among the tested EOs, CIN exhibited the most selective profile—eliciting strong attraction on *S. oryzae*, while having significant repellent effects on *L. distinguendus*. In contrast, the other two EOs (NM and CL) did not demonstrate statistically significant attractiveness towards *S. oryzae*, though they showed a trend toward attracting the pest and also demonstrated statistically significant repellency towards *L. distinguendus*. These findings highlight the potential of CIN as a selective tool in stored cereal protection, but its efficacy and compatibility should be validated under real storage conditions. However, laboratory assays, while controlled and reproducible, could not fully replicate the complexity of real storage environments, where airflow, odor dispersion, and the presence of multiple cues can all modulate insect behavior [50]. A limitation of our study is the reliance on laboratory-based behavioral assays, which, while informative, may not capture the full complexity of the real-world storage environments. Thus, further field-based studies are recommended to validate these laboratory findings and to optimize the application of CIN and other EOs for practical use in biological control programs for grain storage [48].

## Figures and Tables

**Table 1:** Complete chemical composition of EOs obtained from *Myristica fragrans* (nutmeg), *Cinnamomum verum* (cinnamon), and *Syzygium aromaticum* (clove).

Compounds	LRI ^1^	LRI ^2^	Class	Relative Abundance ± Standard Deviation		
				Nutmeg	Cinnamon	Clove
α-thujene	922	933	mh	1.1 ± 0.02	- ^3^	-
α-pinene	933	941	mh	17.0 ± 0.60	0.1 ± 0.02	-
camphene	955	955	mh	0.3 ± 0.01	-	-
benzaldehyde	959	962	nt	-	0.1 ± 0.01	-
sabinene	974	976	mh	14.1 ± 0.45	-	-
β-pinene	977	975	mh	13.4 ± 0.42	-	-
myrcene	991	991	mh	1.0 ± 0.03	-	-
α-phellandrene	1006	1004	mh	0.2 ± 0.00	0.3 ± 0.06	-
δ-3-carene	1011	1012	mh	1.0 ± 0.03	-	-
α-terpinene	1017	1020	mh	0.4 ± 0.01	0.2 ± 0.03	-
*p*-cymene	1024	1027	mh	4.0 ± 0.10	0.7 ± 0.12	-
limonene	1029	1031	mh	4.2 ± 0.09		-
β-phellandrene	1029	1032	mh	-	0.9 ± 0.15	-
1,8-cineole	1032	1035	om	0.2 ± 0.01	-	-
2-heptyl acetate	1045	1043	nt	-	-	0.1 ± 0.02
γ-terpinene	1058	1062	mh	0.8 ± 0.04	-	-
*cis*-sabinene hydrate	1066	1066	om	0.3 ± 0.00	-	-
terpinolene	1089	1088	mh	0.5 ± 0.01	-	-
*trans*-sabinene hydrate	1098	1099	om	0.3 ± 0.02	-	-
linalool	1101	1101	om	0.2 ± 0.01	1.4 ± 0.22	-
hydroxycinnamaldehyde	1162	-	pp	-	0.3 ± 0.03	-
4-terpineol	1177	1179	om	5.0 ± 0.02	0.1 ± 0.02	-
*p*-cymen-8-ol	1185	1185	om	0.3 ± 0.01	-	-
α-terpineol	1191	1191	om	-	0.3 ± 0.04	-
methyl salicylate	1194	1192	nt	-	-	0.3 ± 0.02
(*Z*)-cinnamaldehyde	1219	1219	pp	-	0.3 ± 0.01	-
(*E*)-cinnamaldehyde	1270	1270	pp	-	78.5 ± 1.58	-
*trans*-ascaridol glycol	1271	1273	om	0.2 ± 0.00	-	-
carvacrol	1302	1300	om	0.2 ± 0.01	-	-
eugenol	1356	1359	pp	-	5.2 ± 0.43	89.2 ± 0.2
α-copaene	1376	1377	sh	-	0.6 ± 0.13	-
methyl eugenol	1405	1402	pp	0.2 ± 0.02	-	-
β-caryophyllene	1419	1420	sh	-	4.3 ± 0.97	3.9 ± 0.04
(*E*)-cinnamyl acetate	1449	1448	pp	-	3.8 ± 0.05	
α-humulene	1453	1455	sh	-	0.8 ± 0.18	0.5 ± 0.01
(*E*)-methyl isoeugenol	1492	1495	pp	0.1 ± 0.00	-	-
(*E*)-*o*-methoxycinnamaldehyde	1505	1505	pp	-	0.4 ± 0.14	-
myristicin	1521	1520	pp	33.0 ± 1.59	-	-
eugenol acetate	1528	1525	pp	-	-	5.8 ± 0.14
elemicin	1558	1559	pp	1.8 ± 0.10	-	-
caryophyllene oxide	1581	1581	os	-	0.5 ± 0.12	0.2 ± 0.02
tetradecanal	1613	1611	nt	-	0.2 ± 0.04	-
benzyl benzoate	1763	1762	nt	-	1.1 ± 0.04	-
Monoterpene hydrocarbons (mh)				58.0 ± 1.78	2.2 ± 0.37	-
Oxygenated monoterpenes (om)				6.7 ± 0.04	1.8 ± 0.28	-
Sesquiterpene hydrocarbons (sh)				-	5.7 ± 1.28	4.4 ± 0.05
Oxygenated sesquiterpenes (os)				-	0.5 ± 0.12	0.2 ± 0.02
Phenylpropanoids (pp)				35.1 ± 1.71	88.5 ± 2.11	95.0 ± 0.06
Other non-terpene derivatives (nt)				-	1.4 ± 0.08	0.4 ± 0.00
Total identified (%)				99.8 ± 0.04	100.1 ± 0.01	100.0 ± 0.00

^1^ Linear retention index on a HP-5MS capillary column. ^2^ LRI values from the literature (Adams, 2007; NIST Chemistry WebBook) [29]. ^3^ Not detected.

**Table 2:** Results of behavior of *Sitophilus oryzae* adults in the area preference method (APM) exposed to three essential oils (EOs) at concentrations of 0.94–3.77 µL L^−1^ air equivalent. Data are expressed as mean Preference Index (PI) ± standard deviation (SD). PI < −0.11 indicates repellent; −0.10 ≤ PI ≤ 0.10 indicates neutral, and PI > 0.11 indicates attractive (from Lacotte et al.) [30].

**EO**	**Preference Index (PI; Mean ± SD)**		
	**Concentration of EO (µL L^−1^ of Air Equivalent)**		
	0.94	1.89	3.77
Cinnamon	0.38 ± 0.16 ^A^	0.40 ± 0.23 ^A^	0.50 ± 0.16 ^A^
Clove	0.40 ± 0.21 ^A^	0.48 ± 0.16 ^A^	0.56 ± 0.11 ^A^
Nutmeg	0.14 ± 0.11 ^B^	0.24 ± 0.18 ^B^	0.22 ± 0.19 ^B^

^A–B^ Different superscript letters within a column indicate significant differences among EO treatments according to Duncan’s multiple range test (*p* < 0.05).

**Table 3:** Behavioral responses of *Sitophilus oryzae* adults to major constituents of EOs, assessed in Area Preference Method (APM) across three concentrations (0.94, 1.89, and 3.77 µL L^−1^ air equivalent). Data are expressed as mean Preference Index (PI) ± standard deviation (SD). Color coding: orange = repellent (PI < −0.11), white = neutral response (−0.10 ≤ PI ≤ 0.10), green = attractive (PI > 0.11), following thresholds adapted from Lacotte et al. [30]. EO abbreviations: CIN = cinnamon, CL = clove, NM = nutmeg.

**Pure Component**	**Originating Essential Oil**	**Preference Index (PI; Mean ± SD)**		
		**Concentration of Pure Components** **(µL L^−1^ Air Equivalent)**		
		0.94	1.89	3.77
Myristicin	NM	0.73 ± 0.12	0.10 ± 0.26	−0.33 ± 0.25
β-pinene	NM	0.40 ± 0.34	−0.02 ± 0.18	−0.18 ± 0.43
α-pinene	CIN, NM	0.38 ± 0.18	0.04 ± 0.62	−0.26 ± 0.15
Limonene	NM	0.20 ± 0.10	0.08 ± 0.23	−0.20 ± 0.07
Cinnamaldehyde	CIN	0.16 ± 0.24	−0.16 ± 0.27	−0.20 ± 0.16
β-caryophyllene	CIN, CL	0.02 ± 0.50	−0.16 ± 0.23	−0.24 ± 0.17
1,8-cineole	NM	−0.02 ± 0.13	0.04 ± 0.19	−0.14 ± 0.11
Sabinene	NM	−0.06 ± 0.30	−0.16 ± 0.18	−0.22 ± 0.43
Terpinolene	NM	−0.14 ± 0.09	−0.26 ± 0.17	−0.36 ± 0.21
Linalool	NM, CIN	−0.18 ± 0.08	−0.30 ± 0.22	−0.24 ± 0.23
Methyl eugenol	NM	−0.26 ± 0.05	−0.26 ± 0.11	−0.36 ± 0.11
α-terpineol	CIN	−0.48 ± 0.31	−0.48 ± 0.27	−0.50 ± 0.07
Eugenol	CIN, CL	−0.58 ± 0.33	−0.66 ± 0.18	−0.70 ± 0.10
**Positive Control**				
MR-08	-	−0.18 ± 0.26	−0.26 ± 0.09	−0.42 ± 0.19

## Data Availability

The original contributions presented in the study are included in the article, further inquiries can be directed to the corresponding authors.

## Abbreviations

The following abbreviations are used in this manuscript:

APM	Area Preference Method
CIN	Cinnamon oil (*Cinnamomum verum*)
CL	Clove oil (*Syzygium aromaticum*)
EOs	Essential oils
GC-MS	Gas Chromatography–Mass Spectrometry
LRI	Linear Retention Index
NM	Nutmeg oil (*Myristica fragrans*)
PI	Preference Index
RH	Relative Humidity
SD	Standard Deviation
TCB	Two-Choice Behavioral Bioassay

## References

[1] Food and Agriculture Organization of the United Nations (2023). World Food and Agriculture–Statistical Yearbook 2023.

[2] Kumar D., Kalita P. (2017). Reducing postharvest losses during storage of grain crops to strengthen food security in developing countries. Foods.

[3] Berhe M., Subramanyam B., Chichaybelu M., Demissie G., Abay F., Harvey J. (2022). Post-harvest insect pests and their management practices for major food and export crops in East Africa: An Ethiopian case study. Insects.

[4] Stathas I.G., Sakellaridis A.C., Papadelli M., Kapolos J., Papadimitriou K., Stathas G.J. (2023). The effects of insect infestation on stored agricultural products and the quality of food. Foods.

[5] Daglish G.J., Nayak M.K., Arthur F.H., Athanassiou C.G., Athanassiou C.G., Arthur F.H. (2018). Insect pest management in stored grain. Recent Advances in Stored Product Protection.

[6] Swamy K.C.N., Mutthuraju G.P., Jagadeesh E., Thirumalaraju G.T. (2014). Biology of *Sitophilus oryzae* (L.) (Coleoptera: Curculionidae) on stored maize grains. Curr. Biotica.

[7] Phillips T.W., Jiang X.L., Burkholder W.E., Phillips J.K., Tran H.Q. (1993). Behavioral responses to food volatiles by two species of stored-product coleoptera, *Sitophilus oryzae* (curculionidae) and *Tribolium castaneum* (tenebrionidae). J. Chem. Ecol..

[8] Sharifi S., Mills R.B. (1971). Developmental activities and behavior of the rice weevil inside wheat kernels. J. Econ. Entomol..

[9] Soujanya P.L., Sekhar J.C., Karjagi C.G., Vidhyadhari V., Suby S.B., Sunil N., Sreelatha D., Chaudhary D. (2017). Impact of harvesting time on field carry over infestation of *Sitophilus oryzae* (L.) (Coleoptera: Curculionidae) in different maize genotypes. Phytoparasitica.

[10] Agha M.K., Lee W., Wang C., Mankin R., Blount A., Bucklin R., Bliznyuk N. (2017). Detection and prediction of *Sitophilus oryzae* infestations in triticale via visible and near-infrared spectral signatures. J. Stored Prod. Res..

[11] Imura O., Sinha R.N. (1984). Effect of infestation by *Sitotroga cerealella* (Lepidoptera: Gelechiidae) and *Sitophilus oryzae* (Coleoptera: Curculionidae) on the deterioration of bagged wheat. Environ. Entomol..

[12] DiBartolomeis M., Kegley S., Mineau P., Radford R., Klein K. (2019). An assessment of acute insecticide toxicity loading (AITL) of chemical pesticides used on agricultural land in the United States. PLoS ONE.

[13] Nicolopoulou-Stamati P., Maipas S., Kotampasi C., Stamatis P., Hens L. (2016). Chemical pesticides and human health: The urgent need for a new concept in agriculture. Front. Public Health.

[14] Adarkwah C., Obeng-Ofori D., Büttner C., Reichmuth C., Schöller M. (2011). Potential of *Lariophagus distinguendus* (Förster) (Hymenoptera: Pteromalidae) to suppress the maize weevil *Sitophilus zeamais* Motschulsky (Coleoptera: Curculionidae) in bagged and bulk stored maize. Biol. Control.

[15] Benelli G., Pacini N., Conti B., Canale A. (2013). Following a scented beetle: Larval faeces as a key olfactory cue in host location of *Stegobium paniceum* (Coleoptera: Anobiidae) by *Lariophagus distinguendus* (Hymenoptera: Pteromalidae). Chemoecology.

[16] Niedermayer S., Pollmann M., Steidle J. (2016). *Lariophagus distinguendus* (Hymenoptera: Pteromalidae) (Förster)—Past, present, and future: The history of a biological control method using *L. distinguendus* against different storage pests. Insects.

[17] Fields P.G., White N.D.G. (2002). Alternatives to methyl bromide treatments for stored-product and quarantine insects. Annu. Rev. Entomol..

[18] Hu Z., Yang J., Chen Z., Chang C., Ma Y., Li N., Deng M., Mao G., Bao Q., Deng S. (2022). Exploration of clove bud (*Syzygium aromaticum*) essential oil as a novel attractant against *Bactrocera dorsalis* (Hendel) and Its safety evaluation. Insects.

[19] Tian Y., Hogsette J.A., Norris E.J., Hu X.P. (2024). Topical toxicity and repellency profiles of 17 essential oil components against insecticide-resistant and susceptible strains of adult *Musca domestica* (Diptera: Muscidae). Insects.

[20] Gupta I., Singh R., Muthusamy S., Sharma M., Grewal K., Singh H.P., Batish D.R. (2023). Plant essential oils as biopesticides: Applications, mechanisms, innovations, and constraints. Plants.

[21] Maurya A., Prasad J., Das S., Dwivedy A.K. (2021). Essential oils and their application in food safety. Front. Sustain. Food Syst..

[22] Turek C., Stintzing F.C. (2013). Stability of essential oils: A review. Compr. Rev. Food Sci. Food Saf..

[23] Parichanon P., Ascrizzi R., Echeverría M.C., Farina P., Pieracci Y., Flamini G., Semprucci F., Guidi L., Grassi E., Gogou T.I. (2025). The aromatic plant essential oils and their hormetic effect on *Rhyzopertha dominica* (Coleoptera: Bostrichidae). Crop Prot..

[24] Bedini S., Djebbi T., Ascrizzi R., Farina P., Pieracci Y., Echeverría M.C., Flamini G., Trusendi F., Ortega S., Chiliquinga A. (2024). Repellence and attractiveness: The hormetic effect of aromatic plant essential oils on insect behavior. Ind. Crops Prod..

[25] Zeni V., Benelli G., Campolo O., Giunti G., Palmeri V., Maggi F., Rizzo R., Lo Verde G., Lucchi A., Canale A. (2021). Toxics or lures? Biological and behavioral effects of plant essential oils on tephritidae fruit flies. Molecules.

[26] Zhan H., Li L., Li F., Zang L. (2023). Identification and comparative expression profiles of candidate olfactory receptors in the transcriptomes of the important egg parasitoid wasp *Anastatus japonicus* Ashmead (Hymenoptera: Eupelmidae). Plants.

[27] Ruther J., Schmitt T., Stökl J. (2023). Editorial: Recent advances in the chemical ecology of parasitic Hymenoptera. Front. Ecol. Evol..

[28] Cai P., Song Y., Huo D., Lin J., Zhang H., Zhang Z., Xiao C., Huang F., Ji Q. (2020). Chemical cues induced from fly-oviposition mediate the host-seeking behaviour of *Fopius arisanus* (Hymenoptera: Braconidae), an effective egg parasitoid of *Bactrocera dorsalis* (Diptera: Tephritidae), within a Tritrophic Context. Insects.

[29] Adams R.P. (2007). Identification of Essential Oil Components by Gas Chromatography/Mass Spectrometry.

[30] Lacotte V., Rey M., Peignier S., Mercier P., Rahioui I., Sivignon C., Razy L., Benhamou S., Livi S., Da Silva P. (2023). Bioactivity and chemical composition of forty plant essential oils against the pea aphid *Acyrthosiphon pisum* revealed peppermint oil as a promising biorepellent. Ind. Crops Prod..

[31] Abenaim L., Mandoli A., Venturi F., Bedini S., Conti B. (2022). Evaluation of a quasi-dimeric eugenol derivative as repellent against the stored grain insect pest *Sitophilus oryzae* (Coleoptera: Curculionidae). Pest Manag. Sci..

[32] Ninkovic V., Markovic D., Rensing M. (2020). Plant volatiles as cues and signals in plant communication. Plant Cell Environ..

[33] Deletre E., Chandre F., Williams L., Duménil C., Menut C., Martin T. (2015). Electrophysiological and behavioral characterization of bioactive compounds of the *Thymus vulgaris*, *Cymbopogon winterianus*, *Cuminum cyminum* and *Cinnamomum zeylanicum* essential oils against *Anopheles gambiae* and prospects for their use as bednet treatments. Parasites Vectors.

[34] Sarma R., Adhikari K., Mahanta S., Khanikor B. (2019). Combinations of plant essential oil based terpene compounds as larvicidal and adulticidal agent against *Aedes aegypti* (Diptera: Culicidae). Sci. Rep..

[35] De Brito Sanchez M.G., Lorenzo E., Su S., Liu F., Zhan Y., Giurfa M. (2014). The tarsal taste of honey bees: Behavioral and electrophysiological analyses. Front. Behav. Neurosci..

[36] Galizia C.G., Rössler W. (2009). Parallel olfactory systems in insects: Anatomy and function. Annu. Rev. Entomol..

[37] Akotsen-Mensah C., Blaauw B.R., Rivera M.J., Rodriguez-Saona C., Nielsen A.L. (2021). Behavioral response of *Halyomorpha halys* (Hemiptera: Pentatomidae) and its egg parasitoid *Trissolcus japonicus* (Hymenoptera: Scelionidae) to host plant odors. Front. Ecol. Evol..

[38] Ahmed Q., Agarwal M., Al-Obaidi R., Wang P., Ren Y. (2021). Evaluation of aphicidal effect of essential oils and their synergistic effect against *Myzus persicae* Sulzer (Hemiptera: Aphididae). Molecules.

[39] Filomeno C.A., Barbosa L.C.A., Teixeira R.R., Pinheiro A.L., De Sá Farias E., Ferreira J.S., Picanço M.C. (2020). Chemical diversity of essential oils of Myrtaceae species and their insecticidal activity against *Rhyzopertha dominica*. Crop Prot..

[40] Giunti G., Benelli G., Palmeri V., Laudani F., Ricupero M., Ricciardi R., Maggi F., Lucchi A., Guedes R.N.C., Desneux N. (2022). Non-target effects of essential oil-based biopesticides for crop protection: Impact on natural enemies, pollinators, and soil invertebrates. Biol. Control.

[41] Sulg S., Kaasik R., Kallavus T., Veromann E. (2023). Toxicity of essential oils on cabbage seedpod weevil (*Ceutorhynchus obstrictus*) and a model parasitoid (*Nasonia vitripennis*). Front. Agron..

[42] Yuan J.S., Köllner T.G., Wiggins G., Grant J., Degenhardt J., Chen F. (2008). Molecular and genomic basis of volatile-mediated indirect defense against insects in rice. Plant J..

[43] Cao Y., Hu Q., Huang L., Athanassiou C.G., Maggi F., D’Isita I., Liu Y., Pistillo O.M., Miao M., Germinara G.S. (2023). Attraction of *Sitophilus oryzae* (L.) (Coleoptera: Curculionidae) to the semiochemical volatiles of stored rice materials. J. Pest Sci..

[44] Tang Q., Yang T., Jiang J. (2016). Herbivore-induced rice grain volatiles affect attraction behavior of herbivore enemies. Interciencia.

[45] Wright G.A. (2004). Different thresholds for detection and discrimination of odors in the honey bee (*Apis mellifera*). Chem. Senses.

[46] Ayllón-Gutiérrez R., Díaz-Rubio L., Montaño-Soto M., Del Pilar Haro-Vázquez M., Córdova-Guerrero I. (2024). Applications of plant essential oils in pest control and their encapsulation for controlled release: A review. Agriculture.

[47] Cook S.M., Khan Z.R., Pickett J.A. (2006). The use of push-pull strategies in integrated pest management. Annu. Rev. Entomol..

[48] Navarro-Llopis V., Alfaro F., Domínguez J., Sanchis J., Primo J. (2008). Evaluation of traps and lures for mass trapping of Mediterranean fruit fly in citrus groves. J. Econ. Entomol..

[49] Witzgall P., Kirsch P., Cork A. (2010). Sex pheromones and their impact on pest management. J. Chem. Ecol..

[50] Isman M.B. (2019). Botanical insecticides in the twenty-first century—Fulfilling their promise?. Annu. Rev. Entomol..

[51] Bell C. (2014). A review of insect responses to variations encountered in the managed storage environment. J. Stored Prod. Res..

[52] Leal W.S. (2013). Odorant reception in insects: Roles of receptors, binding proteins, and degrading enzymes. Annu. Rev. Entomol..

[53] Venthur H., Zhou J. (2018). Odorant receptors and odorant-binding proteins as insect pest control Targets: A comparative analysis. Front. Physiol..

[54] National Institute of Standards and Technology (NIST) (2014). NIST/EPA/NIH Mass Spectral Library (NIST Standard Reference Database Number 69).

[55] Zimmermann R.C., de Carvalho Aragão C.E., de Araújo P.J.P., Benatto A., Chaaban A., Martins C.E.N., Zawadneak M.A.C. (2021). Insecticide activity and toxicity of essential oils against two stored-product insects. Crop Prot..

[56] Lee H., Hong S.J., Hasan N., Baek E.J., Kim J.T., Kim Y., Park M. (2020). Repellent efficacy of essential oils and plant extracts against *Tribolium castaneum* and *Plodia interpunctella*. Entomol. Res..

[57] Caballero-Gallardo K., Fuentes-Lopez K., Stashenko E.E., Olivero-Verbel J. (2023). Chemical composition, repellent action, and toxicity of essential oils from *Lippia origanoides*, *Lippia alba* chemotypes, and *Pogostemon cablin* on adults of *Ulomoides dermestoides* (Coleoptera: Tenebrionidae). Insects.

